# Facing the Heat: Does Desiccation and Thermal Stress Explain Patterns of Orientation in an Intertidal Invertebrate?

**DOI:** 10.1371/journal.pone.0150200

**Published:** 2016-03-09

**Authors:** Clarissa M. L. Fraser, Frank Seebacher, Justin Lathlean, Ross A. Coleman

**Affiliations:** 1 Centre for Research on Ecological Impacts of Coastal Cities, School of Biological Sciences, The University of Sydney, Sydney, New South Wales, Australia; 2 School of Biological Sciences, The University of Sydney, Sydney, New South Wales, Australia; 3 School of Biological Sciences, University of Wollongong, Wollongong, New South Wales, Australia; 4 School of Biological, Earth and Environmental Sciences, University of New South Wales, Sydney, New South Wales, Australia; Australian Institute of Marine Science, AUSTRALIA

## Abstract

A key challenge for ecologists is to quantify, explain and predict the ecology and behaviour of animals from knowledge of their basic physiology. Compared to our knowledge of many other types of distribution and behaviour, and how these are linked to individual function, we have a poor level of understanding of the causal basis for orientation behaviours. Most explanations for patterns of animal orientation assume that animals will modify their exposure to environmental factors by altering their orientation. We used a keystone grazer on rocky shores, the limpet *Cellana tramoserica*, to test this idea. Manipulative experiments were done to evaluate whether orientation during emersion affected limpet desiccation or body temperature. Body temperature was determined from infrared thermography, a technique that minimises disturbance to the test organism. No causal relationships were found between orientation and (i) level of desiccation and (ii) their body temperature. These results add to the growing knowledge that responses to desiccation and thermal stress may be less important in modifying the behaviour of intertidal organisms than previously supposed and that thermoregulation does not always reflect patterns of animal orientation. Much of what we understand about orientation comes from studies of animals able to modify orientation over very short time scales. Our data suggests that for animals whose location is less flexible, orientation decisions may have less to do with responses to environmental factors and more to do with structural habitat properties or intrinsic individual attributes. Therefore we suggest future studies into processes affecting orientation must include organisms with differing levels of behavioural plasticity.

## Introduction

Physiological constraints have often been used to explain small-scale variations in the behaviour and distribution of animals [1–4]. Orientation, the fine-scale position with respect to a directional stimulus of an animal in space at a given point in time, varies greatly among individuals and at different temporal and spatial scales (nightingales [5], black wildebeest [6], spiders [7]). Across a wide array of taxa, associations have been established between an individual’s orientation and environmental variables (e.g. [6, 8–11]). By varying the surface area exposed to the stimulus of interest, frequently the sun, wind or waves, individuals can alter the effect of this stimulus on their body (e.g. [12, 13, 14]). Establishing causal links between orientation and potential benefits or costs for individuals, such as differences in mating success or physiological responses, is the obvious next step towards understanding the importance of orientation has for individuals and/or fitness.

Biases in orientation are commonly thought to be associated with thermoregulation (e.g. [8, 10, 14, 15, 16, 17]). Biophysical modelling has suggested that differences in body orientation with respect to solar radiation and wind direction can alter body temperature by up to 18°C and 1°C, respectively, in a 1 kg terrestrial ectotherm [3]. Similarly, male killer wasps *Sphecius speciosus* orientate perpendicular to the sun during cooler parts of the day and are then parallel to solar radiation during hotter periods; when dead conspecifics were manipulated to face different directions, operative temperatures were greater in perpendicularly aligned individuals than those placed parallel [16]. Manipulative experiments in the field are the ideal approach to test predications about causal relationships between behaviour and ecological physiology [18] but, in the context of orientation, it is difficult to manipulate highly mobile organisms. Additionally, experimental findings regarding the role of orientation in thermoregulation are contradictory, and some studies show that orientation does not influence body temperature as predicted (e.g in lizards[11], [19], butterflies [20]). Generalisation across taxa is therefore premature and further manipulative experiments are needed, preferably in the field.

Responses to thermal stress and/or desiccation stress are two of the most frequently proposed underlying explanations for observed patterns of abundance, distribution and behaviour of intertidal organisms (e.g. [1, 2, 21–24]). Compared to the volumes of experimental research on desiccation, thermal stress and the distribution of intertidal gastropods, possible links between orientation and potential physiological stresses are much less investigated. Most studies of orientation in the rocky intertidal environment have focused on thermoregulation in littorinids [14, 25–27]. For example, in the South American periwinkle *Echinolittorina peruviana*, the majority of individuals position their body to face the sun dorso-ventrally, as opposed to laterally, to reduce exposure to solar radiation and this pattern is associated with differences in body temperature [14]. The relative importance of orientation in gastropod thermoregulation is however not conclusive [26] since previous studies [14, 27] did not measure body temperature directly, or lacked appropriate procedural controls (e.g.[25]). To increase the generality of findings and reliability of results, studies should be done on organisms other than littorinids and use manipulative field experiments, so enabling tests of causal links [18, 28].

Limpets are an ideal organism for field-based tests of hypotheses on the causal relationships between orientation and physiological and fitness outcomes. This is because their ability to alter their orientation is limited at short temporal scales, only changing when immersed or splashed as the tide rises, this means that limpets can be manipulated more easily than other, more mobile organisms. Limpets are also widely studied, easily accessible and play an integral role in the ecology and structuring of their ecosystem [21, 29–31].

Populations of the intertidal limpet *Cellana tramoserica* exhibit a downwards bias in head orientation on steeply sloped substrata [32]. Proposed explanations for biases in orientation are that a downwards orientation reduces desiccation [33, 34] or thermal stress. These benefits may occur via altering the surface area of the individual to direct solar radiation [3] or ensuring certain parts of the organism are kept moist for longer [33, 35]. Some animals orientate so their head is protected from, via shading, or exposed to solar radiation [e.g. 16]; this may also be true for limpets. Consequently, although overall body temperatures may be similar, anterior temperatures may be different between limpets orientating in different directions.

Here we test the idea that differences in limpet orientation are associated with variations in a) desiccation stress, b) body temperature and/or c) anterior temperature. If there is an absence of any relationship between orientation and desiccation stress it may be because there is no causal link between the two or because limpets that are more sensitive to desiccation compensate by orientating downwards and those which are less sensitive have no need to do so. The same applies for any differences in body or anterior temperature. We performed manipulative experiments, in which the orientation of individuals was altered to measure potential causal effects on desiccation and body temperature. The following hypotheses were tested:

H1) There is a significant correlation between orientation and desiccation stress
H2) Limpets manipulated to face upwards will be, on average, more desiccated then those orientating downwards
H3) Limpets orientating downwards will have, on average, a cooler body and anterior temperature then those orientating upwards
H4) Limpets manipulated to face upwards will, on average, have a hotter body and anterior temperature then those orientating downwards

## Materials and Methods

### Site description

The study was done at two locations near Sydney; Little Bay (033° 58’ S, 151° 15’ E) and Cape Banks Scientific Reserve (034° 59’ S, 151° 25’ E). In New South Wales, scientific research on any marine organism is regulated by a scientific research permit from NSW Department of Primary Industries—Fisheries; called a Fisheries Scientific Research Permit. On this permit, the permissible locations for research, including organism collection, are specified. *C*. *tramoserica* is not an endangered or protected species so no further permits are needed. On our research permit, number F96/146-7.3, Cape Banks Aquatic Reserve and other non-gazetted areas with New South Wales are identified as a permissible locations for research including to collect organisms—research access is controlled under the same permit number. Little Bay is not a gazetted area and no access permits are needed. Patterns of orientation of limpets are similar at each location ([32]; C. Fraser unpub. Data). In all experiments, experimental patches (approx. size 2 x 2m) were haphazardly selected within these locations. Each patch contained steeply-sloped sandstone surfaces (>60°, where 0° is horizontal). Patches were at low- to hightide height above mean low water (the intertidal range of *C*. *tramoserica*) and were moderately exposed to waves [36]. The aspect of surfaces within a patch showed no directional biases and were of mixed rugosity with limited cover of barnacles and macroalgae.

### Measuring orientation

The orientation of limpets was measured using a spirit-level protractor [32] along the anterior-posterior axis of each limpet, where the asymmetric peak of an individual is its head end, and where 0° is normal to gravity.

### Desiccation stress

#### Is there a relationship between orientation and desiccation?

The hypothesis that there is a relationship between limpet orientation and desiccation status (H1) was tested by measuring the orientation of limpets from each of four patches in Cape Banks in January (2 patches, n = 46 and n = 64, maximum air temperature = 32.2°C) and March 2013 (2 patches, n = 89 and n = 85, maximum air temperature = 28.8°C) After a minimum of two hours post low tide, limpets were removed from the substrata using a palette knife, placed in individual sample bags (70mm x 45mm) and transported to the laboratory in a cooled and insulated container. Limpets were processed in the laboratory rather than the field because of logistical and sample size constraints, however the overall effect of this delay in processing has shown to be trivial [37].We measured haemolymph osmolality which is commonly used as an indicator of desiccation stress [1, 22, 37, 38]. Haemolymph osmolality increases with water loss as the relative concentration of compounds in the organism’s haemolymph increases [1]. Limpet haemolymph was collected from the foot as described in Coleman [37] and its osmolality determined using a Wescor Vapour Pressure Osmometer 5500 (Wescor Inc., USA). Triplicate samples were run when possible to increase precision, and the average osmolality value was used as the replicate. Desiccation occurs through evaporative water loss via the gap between shell and substrata [1], and it is therefore a function of shell perimeter length. Shell perimeter was calculated from length and width measurements [37], and the null hypothesis of no relationship between limpet size and desiccation stress was tested using a Pearson’s correlation. Haemolymph was extracted and analysed over two consecutive days, but this had no significant effect on osmolality (S1 File). Instead of testing for a C-linear relationship [39], a relationship between a circular and a linear variable, orientation values for an individual were converted to a difference from 180° and the null hypothesis that there was no linear correlation between orientation and haemolymph osmolality was tested using a Pearson’s correlation for each of the four sites. The Bonferroni method [40] was used to correct α for four repeated tests.

#### Is there a causal link between orientation and desiccation stress?

The orientation of limpets was manipulated in the field on 4 independent sampling dates (maximal air temperatures = 21.8° C, 21.5°C, 28.6°C and 30.9°C on each day respectively) to compare the mean haemolymph osmolality of originally downwards facing limpets that were experimentally changed to face upwards, and limpets which remained unmanipulated and facing downwards (H2). Damage to limpets and water loss from the mantle cavity, as a result of physically removing animals from the substratrum, were avoided by placing limpets on sandstone blocks before the experiment, and then rotating the blocks rather than individuals. Pilot studies have shown that patterns of orientation on the sandstone blocks are similar to those found on natural substrata (% limpets facing downwards on blocks: 40%, on natural substrata: 39%) and limpets appear to experience similar rates of desiccation on both surfaces [41]. The sandstone blocks (200 mm x 200 mm x 30 mm) were attached to existing rocky substrata in the field at least two weeks before the experiment to give sufficient time for biofilm to grow [42]. To control for the possible disturbance effects of unscrewing and rotating the sandstone blocks, some blocks were rotated a full 360 degrees as a procedural control. Blocks were rotated before the limpets were completely emersed but after the animals had ceased foraging movements.

Desiccation stress can increase with the length of time an individual is exposed to air, and because of the limited availability of suitable substrata at the same tidal height to attach our experimental units, the time individuals were exposed to the air varied greatly (80–530 minutes). The time periods during which limpets were emersed was estimated by measuring the time each sandstone block was emersed (+ 5 minutes) from visual monitoring of the blocks. Limpet haemolymph was collected from the foot as above, and the null hypothesis that mean osmolality does not differ between limpets facing different directions was tested using a 2-way General Linear Model in STATISTICA 6 (n = 14) with one fixed factor (Treatment: 3 levels), one random factor (Time: 4 levels) and one covariate, length of time exposed. Heteroscedasticity was tested using Cochran’s *C*-test and the assumption of parallelism of slopes was tested using an homogeneity of slopes model.

### Body and anterior temperature

#### Is there a relationship between body and/or anterior temperature and orientation?

*a) A snapshot of one point in time during emersion*: During October 2013, the hypothesis that there is a difference in the body temperature and/or anterior temperature between upwards and downwards facing limpets (H3) was tested by measuring the body temperature and anterior temperature of limpets in photographs taken across the two locations (Cape Banks and Little Bay, maximal air temperatures 20.9°C and 24.1°C) over two hours. Within each location, 55 pairs of limpets were photographed using a digital thermal imaging camera (Forward looking Infrared S65 ThermaCAM, FLIR, Wilsonville, Oregon USA, thermal resolution 0.08°C; [43]) during low tide, where one limpet was orientated downwards and the other upwards. Shell temperature, as measured by thermal imagery, has previously been shown to accurately predict internal body temperature of limpets, and the following equation was used, to calculate body (BT) and anterior temperature (AT): BT or AT = 1.34 + 1.01ST [41] (S3 File). Visible light photographs were taken of each pair as reference to assist with identifying the limpets in the thermal images. The average body temperature of each limpet was calculated using the program ThermaCAM Researcher Pro 2.10 (FLIR®). Anterior temperature was determined from the average temperature of the area anterior to a line perpendicular to the anterior posterior axis of the limpet positioned across the asymmetric peak of each individual (S1 Fig). Emissivity [44] was set at 0.929 (based on calibrations in a pilot study), and air temperature and humidity settings were based upon weather data collected on the day of sampling. Emissivity is the measure of the thermal radiation emitted by a surface and varies from 0 to 1; it can vary between objects of different colour, surface rugosity and wetness [44]. For each randomly selected pair of limpets (1 ‘upwards’ and 1 ‘downwards’), the difference in body temperature (or anterior temperature) between the two limpets was used as the response variable. The null hypothesis that the mean difference in body temperature (or anterior temperature) did not differ between sites was first tested with a one-way ANOVA (Site: random), before a t-test was done to test the null hypothesis that the mean differences in body or anterior temperatures of upwards and downwards facing limpets were not significantly different from zero. ANOVAs were done in WinGmav 5 (Centre for Research on Ecological Impacts of Coastal Cities, The University of Sydney). The assumptions of homoscedasticity were tested using Cochran’s C-test.

The amount of time an individual is in the sun compared with the shade greatly influences their body temperature [3] and it is possible that differences in body temperature may only occur when in direct sunlight. For each pair of limpets, we monitored the length of time they were exposed to direct sunlight from emersion until they were photographed. Subsequently we tested the null hypothesis that differences in body temperature (or anterior temperature) (n = 6) between upwards and downwards facing limpets did not differ between pairs exposed to sunlight for different lengths of time (100%, 50%, 25% or 0% of the time) using a one-way ANOVA (Exposure time: fixed), as before.

*b) During entirety of emersion*: Snapshot measurements do not always give an accurate picture of thermal stress of intertidal organisms and microhabitat temperatures can vary at small spatial and temporal scales [24, 45, 46]. Hence, we also monitored the temperature of limpets over time to calculate a value that approximates the temperature differently orientated animals experienced over the entirety of low tide. At Little Bay in January 2014 (maximum air temperature = 30.5°C), six pairs of limpets (one downward and upward limpet in each pair) were labelled in each of two patches shortly after the limpets were emersed (approximately 3 hours before low tide) to test the hypothesis that the average body temperature and/or average anterior temperature during the entirety of emersion is different between upwards and downwards facing limpets (H3). Every fifteen minutes for six hours each pair was photographed with a digital thermal imaging camera and the body and anterior temperatures of each limpet were calculated as described above. If temperature measurements are taken at regular intervals, the average of these measurements will be representative of the temperature experienced by each individual. For each pair of limpets, the difference in experienced temperature (as represented by average body or anterior temperature) over the entire period between the two limpets was calculated and used as the response variable. A one-way ANOVA (Site: random) was used to test the null hypothesis that the difference in average body or anterior temperature between paired limpets did not differ between sites. The null hypothesis that the difference in average body or anterior temperature between paired limpets was not different from zero was tested with a t-test.

#### Is there a causal link between orientation and body or anterior temperature?

The hypothesis that mean body and/or anterior temperature of originally downwards facing limpets, rotated to face upwards would be greater than limpets which were unmanipulated and remained facing downwards (H4) was tested using the same procedure as the experiment testing H2. The study was done on two mostly sunny days in April-May 2014 of above average air temperature (maximul air temperature April = 26.4°C and May = 23.4°C, corresponding 30 year averages 23.0°C and 20.3°C, Australian Bureau of Meteorology, www.bom.gov.au). Limpets were photographed one hour before and one hour after low tide using a thermal imaging camera (875i, Testo, Germany, thermal sensitivity 50mK (0.05°C). Body and anterior temperatures of each limpet was determined using the program IRSoft (Version 3.3, Testo AG) and the average body and anterior temperatures from both photographs were calculated as above.

As above, we predicted that differences in anterior and/or body temperature between limpets facing different directions would be found only when limpets were exposed to direct solar radiation, i.e. that there is an interaction between the factors treatment (a limpet’s orientation) and sunlight exposure (described below). On a separate day, we scored exposure to sunlight for each block every 30 minutes, and the total time a block was exposed to sunlight after it was emersed was calculated. Experimental blocks were then divided into two treatments; sunlight exposed (in direct sunlight for a minimum of 70 minutes) and shaded (in shade for entire experimental period). The null hypotheses, that there was no interaction between treatment and sunlight exposure and no difference in body and/or anterior temperature between limpets orientated in different directions, were tested using a 3-way ANOVA (n = 5) with two fixed factors (Treatment: 3 levels, Sun exposed: 2 levels) and one random factor (Time: 2 levels). Data were log transformed when assumptions of homogeneity of variance were not met [47].

## Results

### Desiccation stress

#### Is there a relationship between orientation and desiccation stress?

**Fig 1:**
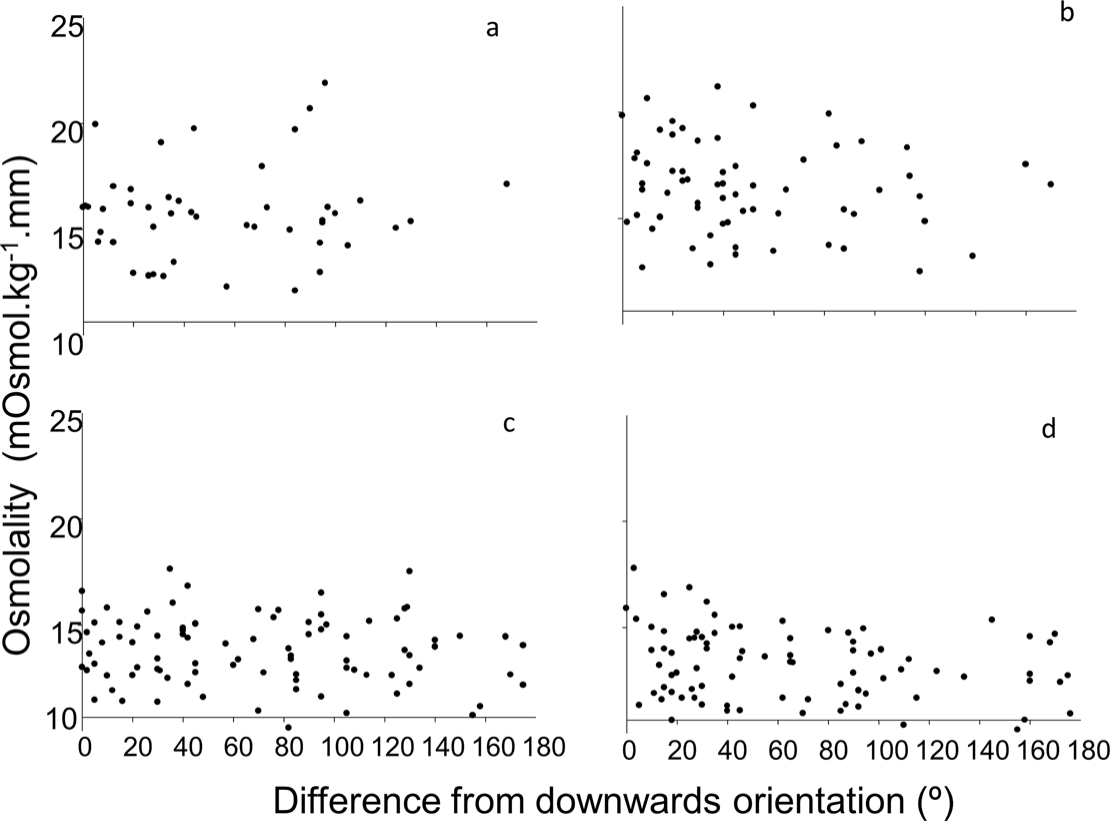
Association between heamolymph osmolality and head orientation (differences in orientation from downwards) in *Cellana tramoserica*. (a) Patch 1 Time 1 n = 46 (b) Patch 2 Time 1 n = 64 (c) Patch 3 Time 2 n = 89 (d) Patch 4 Time 2 n = 85 Downwards is defined as between 135° and 225°, where 0° was straight up [32].

Differences in orientation were not associated with differences in desiccation stress. There was no significant correlation between orientation and haemolymph osmolality (Time 1 Patch 1: r_45_ = 0.080, ns; Time 1 Patch 2: r_63_ = -0.141, ns; Time 2 Patch 3: r_88_ = -0.103, ns; Time 2 Patch 4: r_84_ = -0.251, ns, Fig 1.) and this was consistent across patches and times. There was no relationship between limpet perimeter and osmolality at Time1 (r_109_ = 0.01, ns) but haemolymph osmolality was negatively correlated with perimeter at Time 2 (r_173_ = -0.227, p< 0.05) so all haemolymph osmolality values were standardised for size by dividing the overall osmolality values by shell perimeter.

#### Is there a causal link between orientation and desiccation stress?

**Fig 2:**
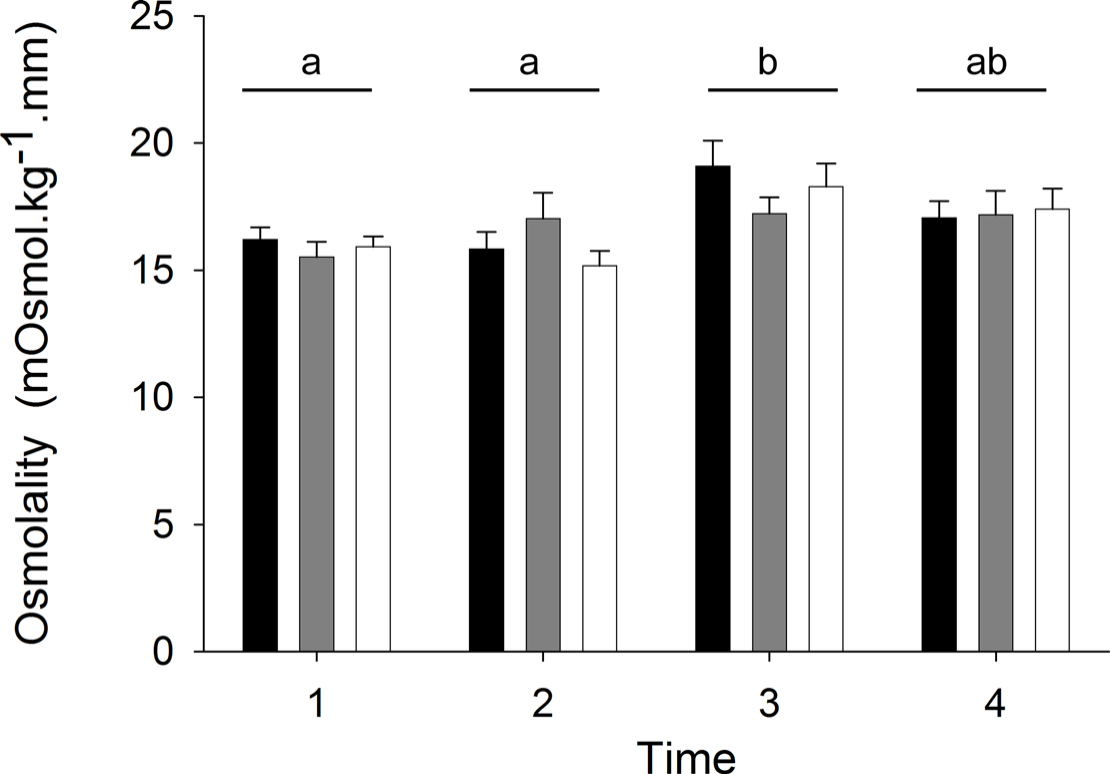
Comparison of mean haemolymph osmolality (+ s.e) between control limpets facing downwards (black bars), limpets rotated 360° (grey bars) and limpets rotated 180° to face upwards (white bars) (n = 14). Lower case numerals denote whether mean osmolality values are significantly different from each other.

No causal link was found between orientation and desiccation stress. The haemolymph osmolality of limpets manipulated to face upwards did not differ from either unmanipulated limpets or limpets rotated 360° (F_(2,6)_ = 0.27, ns, Fig 2., Table B in S2 File). Haemolymph osmolality differed between dates (F_(3,155)_ = 5.20 p<0.05) but the ANCOVA indicated the length of time limpets were exposed had no influence on osmolality (F_(1,155)_ = 0.37, ns).

### Body and anterior temperature

#### Is there a relationship between body and/or anterior temperature and orientation?

**Fig 3:**
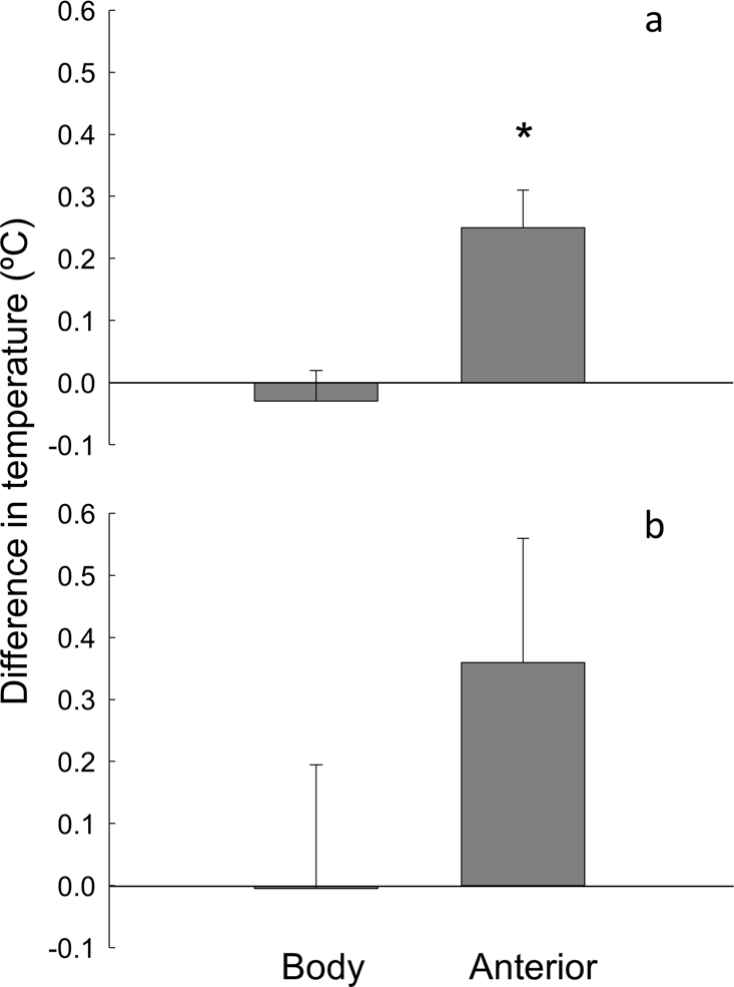
Mean (+ s.e) difference in body and anterior temperature between paired upwards and downwards facing limpets. (a) measured at one point in time (n (combined across locations) = 110 (b) measured during emersion (n (combined across locations) = 12). Difference = temperature of upwards facing limpet–temperature of downwards facing limpet. * = significantly different from 0.

*a) A snapshot at one point in time during emersion*: The data were combined, as patterns were consistent across locations (F_(1,108)_ = 3.79, ns, Table C in S2 File). Orientation and body temperature were not linked, and the mean difference in body temperature between paired upwards and downwards facing limpets was not different from zero (t_109_ = 0.64, ns, Fig 3A). There were significant levels of heteroscedasticity (C = 0.70), which were not stabilised by transformation. Since heteroscedasticity causes an increase in type I errors, this is not a problem for interpretation as the null hypothesis was accepted and no type I error can be made [47]. There was no relationship between time spent in the sun and the difference in body temperature between paired limpets (F_(3,20)_ = 0.13, ns, Table D in S2 File).

Limpet orientation influenced anterior temperature. Differences in anterior temperature between paired upwards and downwards facing limpets were significantly different from zero (t_101_ = 4.19, Fig 3A). Downwards facing limpets were on average 0.25°C cooler than upwards facing limpets. This pattern was consistent across locations (F_(1,100)_ = 0.001, ns, Table E in S2 File) and therefore data were combined. There was significant heteroscedasticity (C = 0.67), which was not fixed by transformation. Since the sample size was relatively large (n = 55) this is less likely to be a type I error [47]. There was no relationship between time spent in the sun and the difference in anterior temperature between paired limpets (F_(3,20)_ = 2.22, ns, Table F in S2 File).

*b) During entirety of emersion*: Orientation was not associated with average body or anterior temperature during emersion; mean differences in average body or anterior temperatures between paired upwards and downwards were not different from zero (Body: t_11_ = 0.02, ns; Anterior: t_11_ = 1.48, ns, Fig 3B). These patterns were consistent across locations (Body: F_(1, 10)_ = 0.001, ns, Table G in S2 File; Anterior: F_(1, 10)_ = 0.08, ns, Table H in S2 File) and therefore data were combined.

#### Is there a causal link between orientation and body or anterior temperature?

**Fig 4:**
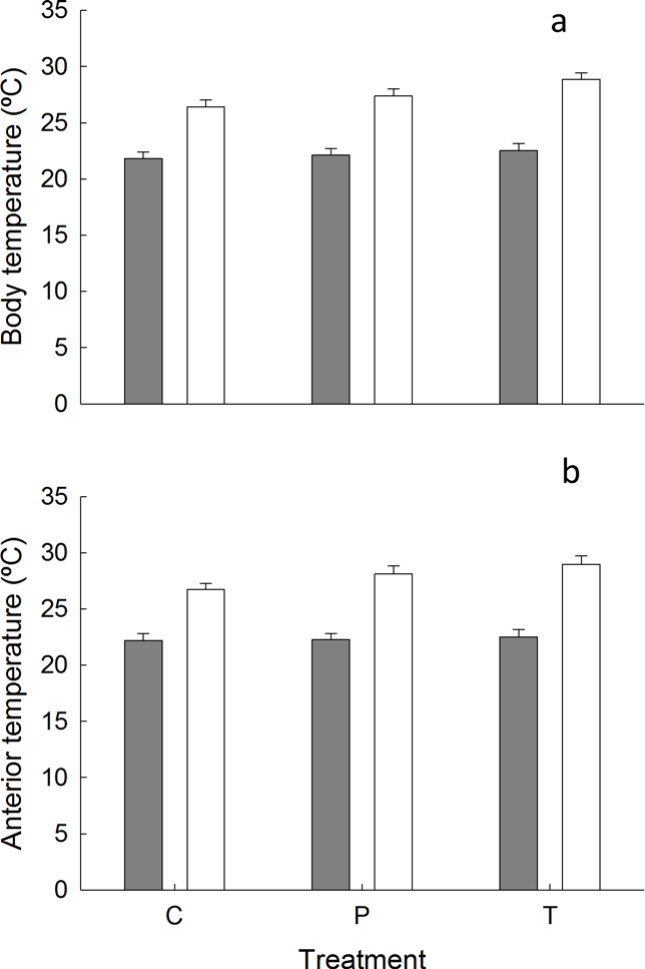
Comparison of mean (+ s.e) body and anterior temperature of limpets orientated in different directions and different habitats (n (combined across sampling times) = 10). (a) body temperature (b) anterior temperature. Treatments were control limpets (C) facing downwards, individuals rotated 360° (P) and individuals rotated 180° to face upwards (T) in the shade (grey bars) and sunlight (white bars).

**Fig 5:**
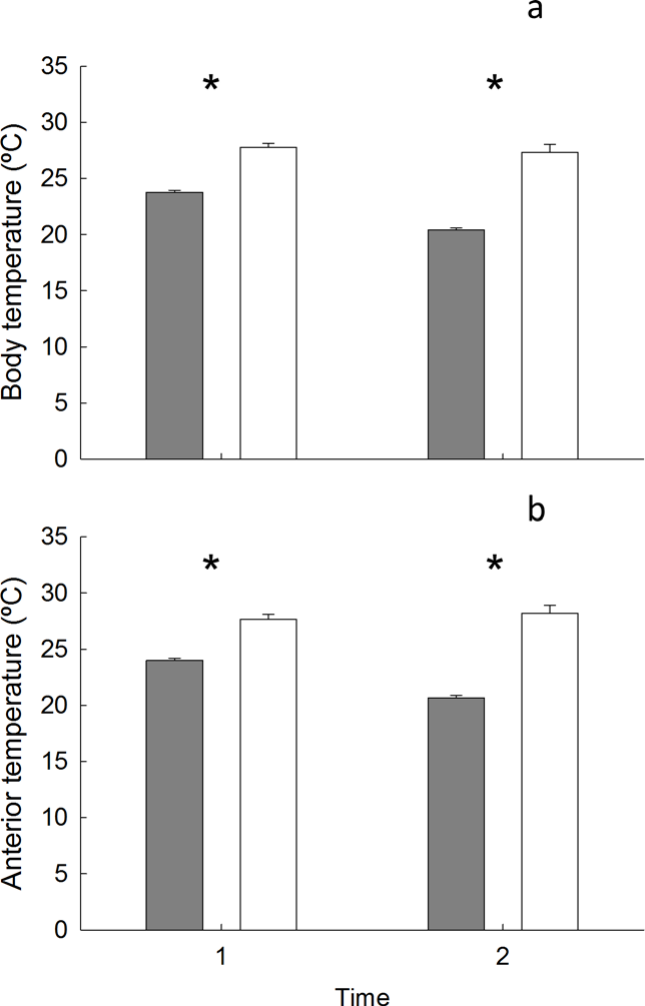
Comparison of mean (s.e) body and anterior temperature of limpets in the shade (grey bars) and sunlight (white bars) (n (combined across treatments) = 15). (a) body temperature (b) anterior temperature. * = significant difference

There was no causal link between body or anterior temperatures and orientation as there was no significant interaction between treatment and sunlight exposure (Body: F_(2,2)_ = 0.75, ns, Fig 4A, Table I in S2 File; Anterior: F_(2,48)_ = 2.24, ns, Fig 4B, Table J in S2 File). Additionally, there were no significant differences in mean body or anterior temperatures between limpets orientated downwards and those rotated 180° (to orientate upwards) or 360° (procedural control) (Body: F_(2,2)_ = 3.98, ns; Anterior: F_(2,48)_ = 2.02, ns). There was a significant interaction between sampling time and whether a limpet was in the sun or shade (Body: F_(1, 48)_ = 16.77, p < 0.05, Fig 5A; Anterior: F_(1, 48)_ = 27.49, p < 0.05, Fig 5B); the magnitude of the differences between body/anterior temperatures in sun and shade differed between the two sampling times. Overall, limpets in the sun were significantly hotter than those in the shade.

## Discussion

The results of each of the mensurative and manipulative experiments (H1 and H2) showed that orientation in *C*. *tramoserica* was not associated with desiccation stress. Haemolymph values were either slightly smaller or equal to those found in physiologically stressed individuals in a congeneric species, *C*. *grata*, but much greater than that of unstressed individuals [22]. In intertidal systems, the effects of desiccation stress, along with thermal stresses and wave exposure, are frequently predicted to explain vertical and along shore patterns of animal distribution (e.g. [1, 2, 21, 22, 48]). Recently manipulative experiments have shown, however, that small-scale patterns of distribution and behaviour, e.g. patterns of aggregation, cannot always be explained by desiccation [37] and our findings concur with this. It is possible that our failure to find a relationship between orientation and desiccation stress is an artefact of the days chosen to test our hypotheses, as maximal daily air temperature varied between sampling dates, and our interpretation would then be a type II error. If this were the case, we would expect a significant interaction between sampling date and experimental treatments, such that the effect of orientation is only manifested on hot days. This did not happen; although desiccation stress overall differed between sampling dates, there were no significant interactions with treatments (Table B in S2 File). Additionally, if the affect of orientation on desiccation was in fact masked by the noise of random variation (from our random factor “sampling date”) then this would suggest that the effect is small and not biologically significant.

Differences in orientation were also not causally linked to either body or anterior temperatures in *C*. *tramoserica* (H4). Although there was a significant difference in anterior temperature between unmanipulated downwards and upwards facing limpets (H3), this only occurred when measured at a single point in time and the difference in anterior temperature was only 0.25°C. This is much smaller than differences observed in other species orientated differently (e.g. 1.9C in robber flies [8], 2.4C in butterflies [20]) or in limpets found in different habitats (e.g. for review see [21], 1.5–3.5°C [22], ~ 5°C [25]) or varying in behaviour (e.g the foot temperature of mushrooming limpets was 2°C cooler [23]). Also, as no difference in anterior temperature was found in the manipulative test of H4, it is reasonable to conclude that there is not likely to be a causal link between anterior temperature and orientation of limpets on steeply sloped rock.

Many studies have found associations between body temperature and orientation [3, 8, 10, 16, 49]. This is in contrast with our findings above, and may be attributed to at least two different models; potential differences in the ability of organisms to alter their orientation in the short term and the contrasting importance of solar radiation in influencing body temperature. In the bulk of organisms whose orientation have been studied, individuals are highly flexible in their ability to alter their orientation relatively quickly as environmental conditions change (e.g. [6, 8, 10, 11]). For example, many species track the position of the sun throughout the day by changing their orientation and thereby altering the surface area they expose to solar radiation (e.g. [8, 16, 50]). In contrast, intertidal limpets, such as *Cellana*, appear to be limited in their ability to alter their orientation during low tide once orientation has been selected. For such animals, the initial orientation decision is more important compared to more mobile organisms, and therefore the behaviour and distribution of individuals prior to orientation selection may be of significantly more importance. In organisms where the initial selection of orientation is more important, external and internal variables, such as structural habitat properties [51–54] and intrinsic individual attributes (for example sex, age and size) [55–57] which are constant during subsequent orientation may have greater importance than environmental factors, which are more likely to change in the same time frame.

The role of direct solar radiation in determining limpet body temperature may be small compared to the effects of convection and conduction. In littorinids, associations between orientation and body temperature exist [14, 25–27]. Littorinids can, however, isolate themselves from the environment by closing their operculum during low tide and therefore solar radiation and convective heat exchange would be of greater importance than conduction [25, 58, 59]. In contrast, limpets always have a large proportion of their foot in contact with the substratum [60–62]. As a consequence, conductive heat exchange is of relatively greater importance in regulating body temperature in limpets compared to solar radiation, although this varies with time of day [63]. Hence, the slope and aspect of the substratum (which partially determines surface temperature) [22, 64, 65], rather than the orientation of individual animals, would have a large effect on body temperature in limpets [63]. A strong relationship between surface temperature and limpet body temperature is supported by the literature [21, 22, 60, 66].

Although thermoregulation is frequently the first, and perhaps most obvious, explanatory model given for patterns of orientation, often other explanations may be needed and the benefits of a specific orientation are not always easily predicated. For instance, differences in orientation with respect to sun position, often assumed to be linked to thermoregulation, are potentially linked to mating success [11, 19, 67]. Although facing downwards may reduce the surface area available to solar radiation, via shading of the anterior end, during the hottest parts of the day (S2 Fig), it also exposes the nuchal cavity to the incoming or outgoing tide and could potentially assist in the removal of waste products such as faecal matter and CO_2_ [35].”The flushing of the nuchal cavity is important as the anus and both the right and left renal openings open into this cavity [61].

In conclusion, we have shown that animal orientation does not always influence thermoregulation and desiccation stress, nor do these stresses consistently drive ecological patterns in intertidal habitats. Variation between species in their ability to alter orientation may lead to differences in the possible drivers and physiological consequences of individual orientation. For animals which are unable to alter their orientation in the short term, environmental conditions, which can fluctuate, may have little influence on orientation compared with habitat properties, which can impact upon initial selection decisions; studies should also now investigate what happens preceding selection in such organisms. Within intertidal gastropods, the importance of orientation in modifying body temperature varies across taxa and for limpets an individual’s location at the scale of the microhabitat appears to be a stronger influence on body temperature. Our results demonstrate the importance of properly designed and controlled manipulative field experiments to test predications about causal links between orientation and individual consequences, and that the use of a less mobile test organism can facilitate this.

## Supporting Information

S1 Fig (PDF)

S2 Fig (PDF)

S1 File (PDF)

S2 File (PDF)

S3 File (PDF)
