# Supplementary material for: Facing the Heat: Does Desiccation and Thermal Stress Explain Patterns of Orientation in an Intertidal Invertebrate?
Source: PLoS One. 2016 Mar 9;11(3):e0150200. doi: 10.1371/journal.pone.0150200 (PMC4784938; doi:10.1371/journal.pone.0150200)
Supplement: S1 Fig — (PDF) [file pone.0150200.s001.pdf]

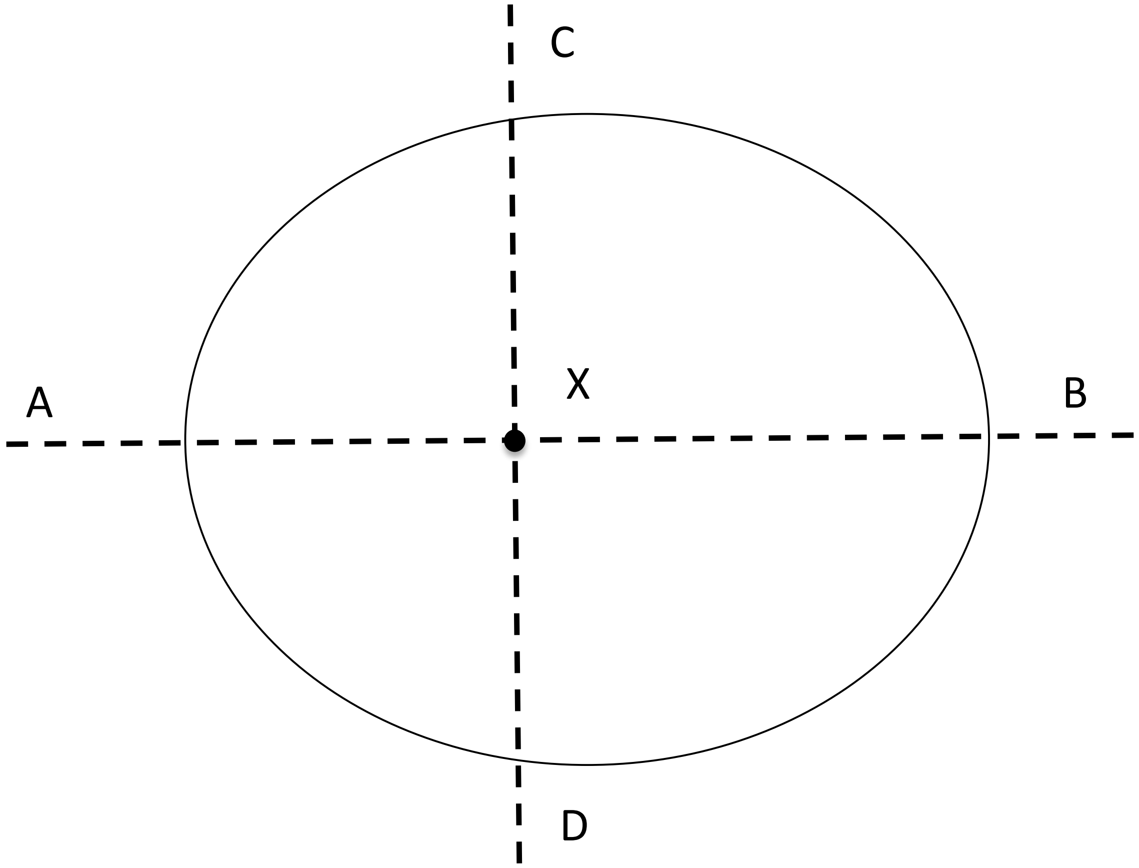


S4 Supplementary Figure. Outline of a typical limpet shell viewed from above. Line A – B represents the anterior –posterior axis with A being the head end. Line C – D is perpendicular to the anterior-posterior axis A-B and crosses it at the peak of the shell, marked X. Anterior part of the shell defined as all parts of the shell anterior of the line C-D
