# Supplementary material for: Facing the Heat: Does Desiccation and Thermal Stress Explain Patterns of Orientation in an Intertidal Invertebrate?
Source: PLoS One. 2016 Mar 9;11(3):e0150200. doi: 10.1371/journal.pone.0150200 (PMC4784938; doi:10.1371/journal.pone.0150200)
Supplement: S2 Fig — (PDF) [file pone.0150200.s002.pdf]

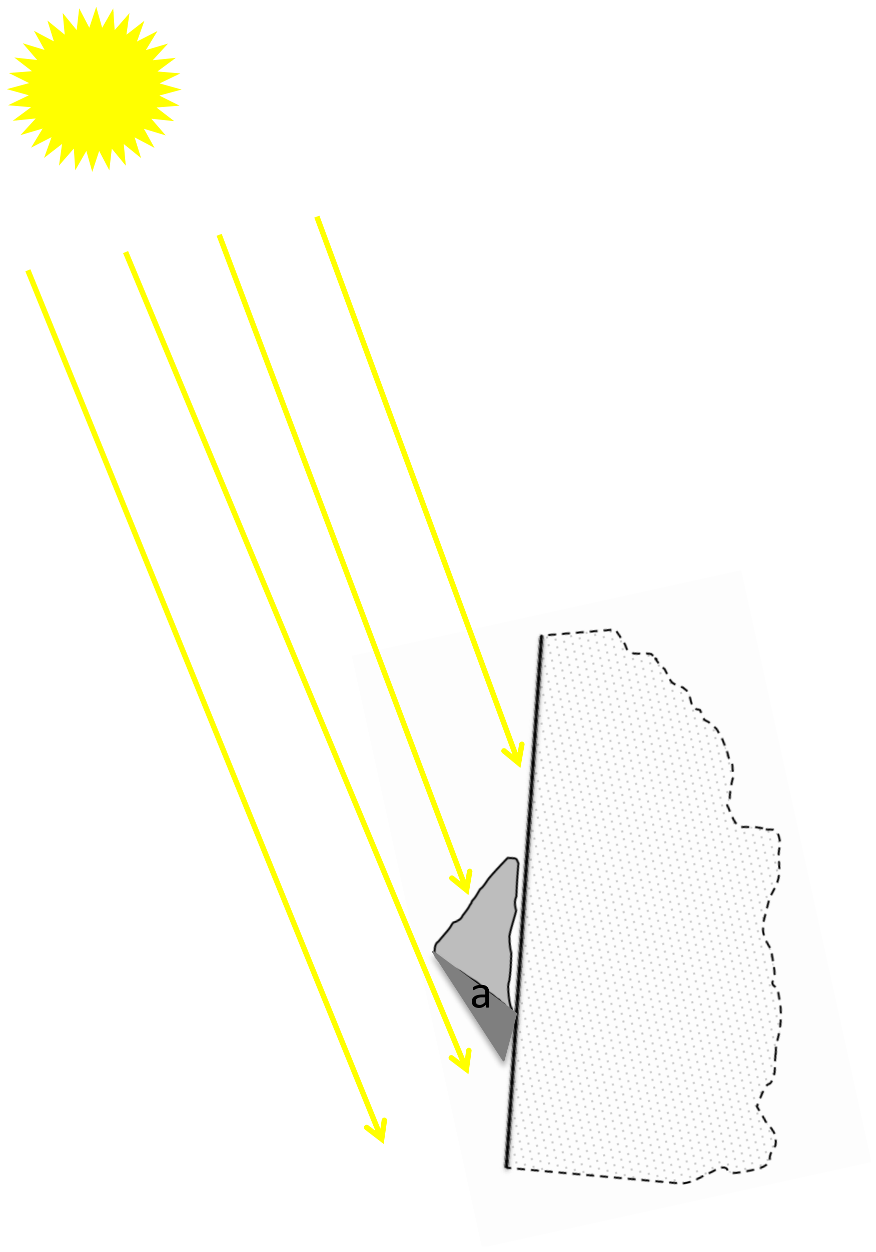

S5 Supplementary Figure. Representation of a limpet orientated downwards exposed to the sun during the hottest part of the day. a: area shaded by asymmetric peak
