# Supplementary material for: Facing the Heat: Does Desiccation and Thermal Stress Explain Patterns of Orientation in an Intertidal Invertebrate?
Source: PLoS One. 2016 Mar 9;11(3):e0150200. doi: 10.1371/journal.pone.0150200 (PMC4784938; doi:10.1371/journal.pone.0150200)
Supplement: S1 File — (PDF) [file pone.0150200.s003.pdf]

Patterns of orientation in an intertidal invertebrate are not explained by desiccation and thermal stress. Clarissa M. L. Fraser*, Frank Seebacher, Justin Lathlean & Ross A. Coleman

* Centre for Research on Ecological Impacts of Coastal Cities, School of Biological Sciences, Marine Ecology Laboratories (A11), The University of Sydney, NSW 2006, AUSTRALIA. Tel. +61 (0)2 9351 4682; Fax +61 (0)2 9351

Supporting Methods

Is there an effect of haemolymph extraction day or sample storage time on osmolality?

Methods

The hypotheses that a) the time taken to extract haemolymph from limpets after collection in the field and b) sample storage time alters haemolymph osmolality were tested by collecting limpets in the field two hours post low tide. Limpets were placed in individual sample bags (70mm x 45mm) and transported to the laboratory in a cooled and insulated container. Limpet haemolymph was collected from the foot as described in Coleman (2010) and its osmolality determined using a Wescor Vapour Pressure Osmometer 5500 (Wescor Inc., USA). Triplicate samples were run when possible to increase precision, and the average osmolality value was used as the replicate. Sample extraction was carried out over two consecutive days, and a one-way ANOVA was run to test the null hypothesis that there was no difference in mean haemolymph osmolality between extraction days. The assumption of heteroscedasticity was tested using Cochran's *C*-test [1]. Previous studies have shown that storing haemolymph samples at 4°C for 1 to 4 days or frozen for 2 weeks has no effect on osmolality [2]. The applicability of this was tested by analysing haemolymph samples over 2 days and a paired t-test was done by resampling a subset of the samples on both days.

Results

The day haemolymph was extracted ($F_{(1,68)} = 0.03$, ns, Table A in S2) and analysed ($t_{35} = 0.24$, ns) had no significant effect on haemolymph osmolality.

References

1. Underwood AJ. Experiments in ecology: their logical design and interpretation using analysis of variance. Cambridge, UK: Cambridge University Press; 1997.

2. Coleman RA. Limpet aggregation does not alter desiccation in the limpet *Cellana tramoserica*. J Exp Mar Biol Ecol. 2010;386(1-2):113-8. doi: 10.1016/j.jembe.2010.02.011.
