# Supplementary material for: Facing the Heat: Does Desiccation and Thermal Stress Explain Patterns of Orientation in an Intertidal Invertebrate?
Source: PLoS One. 2016 Mar 9;11(3):e0150200. doi: 10.1371/journal.pone.0150200 (PMC4784938; doi:10.1371/journal.pone.0150200)
Supplement: S2 File — (PDF) [file pone.0150200.s004.pdf]

Patterns of orientation in an intertidal invertebrate are not explained by desiccation and thermal stress. Clarissa M. L. Fraser*, Frank Seebacher, Justin Lathlean & Ross A. Coleman

* Centre for Research on Ecological Impacts of Coastal Cities, School of Biological Sciences, Marine Ecology Laboratories (A11), The University of Sydney, NSW 2006, AUSTRALIA. Tel. +61 (0)2 9351 4682; Fax +61 (0)2 9351

Table A. Testing the null hypothesis that there was no difference in haemolymph osmolality between limpets whose haemolymph was extracted on different days. There were no significant levels of heteroscedasticity (C = 0.54).

Source	df	MS	F	p
Time Ti	1	0.16	0.03	>0.87
Res	68	4.50		

Table B. Testing the null hypothesis that there is no difference in the haemolymph osmolality of downwards and upwards facing limpets. There was no significant heterogeneity of variance (C = 0.16) and assumptions of parallelism were met ($F_{(6,144)} = 0.22$, ns).

Source	df	MS	F	p
Time exposed	1	0.30	0.04	>0.84
Time Ti	3	41.46	5.20	<0.05
Treatment Tr	2	2.15	0.27	>0.76
Ti X Tr	6	8.18	1.03	>0.41
Res	155	7.97		

Table C. Testing the null hypothesis that there is no difference in the difference in body temperature of downwards and upwards facing limpets between locations.

Source	df	MS	F	p
Location	1	0.95	3.79	>0.05
Res	108	0.25		

Table D. Testing the null hypothesis that there is no difference in the difference in body temperature of downwards and upwards facing limpets in pairs of limpets exposed to sunlight for 0%, 25%, 50% or 100% of the time. There were no significant levels of heteroscedasticity (C = 0.46).

Source	df	MS	F	p
Sunlight Time	3	0.03	0.13	>0.94
Res	20	0.23		

Table E. Testing the null hypothesis that there is no difference in the difference in anterior temperature of downwards and upwards facing limpets between locations.

Source	df	MS	F	p
Location	1	<0.001	<0.00	>0.97
Res	100	0.380		

Table F. Testing the null hypothesis that there is no difference in the difference in anterior temperature of downwards and upwards facing limpets in pairs of limpets exposed to sunlight for 0%, 25%, 50% or 100% of the time. There were no significant levels of heteroscedasticity (C = 0.56).

Source	df	MS	F	p
Sunlight Time	3	0.42	2.22	>0.12
Res	20	0.19		

Table G. Testing the null hypothesis that there is no difference in the difference in body temperature of downwards and upwards facing limpets during emersion between locations. There were no significant levels of heteroscedasticity (C = 0.80).

Source	df	MS	F	p
Location	1	0.002	<0.001	>0.94
Res	10	0.529		

Table H. Testing the null hypothesis that there is no difference in the difference in anterior temperature of downwards and upwards facing limpets during emersion between locations. There were no significant levels of heteroscedasticity (C = 0.61).

Source	df	MS	F	p
Location	1	0.07	0.08	>0.77
Res	10	0.78		

Table I. Testing the null hypothesis that there is no difference in the body temperature of downwards and upwards facing limpets. There were no significant levels of heteroscedasticity in (C = 0.26).

Source	df	MS	F	p
Time Ti	1	54.06	27.66	<0.05
Treatment Tr	2	13.35	3.98	>0.20
Sunlight exposure S	1	444.72	13.57	>0.16
Ti X Tr	2	3.36	1.72	>0.19
Ti X S	1	32.78	16.77	<0.05
Tr X S	2	3.38	0.75	>0.57
Ti X Tr X S	2	4.50	2.30	>0.11
Res	48	1.95		

Table J. Testing the null hypothesis that there is no difference in the anterior temperature of downwards and upwards facing limpets. Data were log transformed to meet assumptions of homogeneity of variance (C = 0.26 after transformation) As there was no significant interaction between treatment, sampling time and sunlight exposure, nor between sampling time and treatment these factors were eliminated following Underwood (1997) to increase the power of the tests above.

Source	df	MS	F	p
Time Ti	1	0.076	18.09	<0.05
Treatment Tr	2	0.009	2.02	>0.14
Sunlight exposure S	1	0.837	7.24	>0.22
Ti X Tr	2	<0.001	* This factor was eliminated	
Ti X S	1	0.116	27.49	<0.05
Tr X S	2	0.009	2.24	>0.11
Ti X Tr X S	2	0.004	* This factor was eliminated	
Res	48	0.004		
