# Supplementary material for: Facing the Heat: Does Desiccation and Thermal Stress Explain Patterns of Orientation in an Intertidal Invertebrate?
Source: PLoS One. 2016 Mar 9;11(3):e0150200. doi: 10.1371/journal.pone.0150200 (PMC4784938; doi:10.1371/journal.pone.0150200)
Supplement: S3 File — (PDF) [file pone.0150200.s005.pdf]

S3 Validation experiment

Relationship between internal body temperature of artificial limpets and shell temperature

The internal body temperature of limpets may not be measured accurately by temperature measurements calculated from thermal images of an individual's shell. To test if shell temperature accurately predicts limpet body temperature, artificial limpets were created and heated to a variety of temperatures before being photographed with a thermal imaging camera (testo 875i thermal imaging camera, Testo, Germany). Artificial limpets were created by embedding iButton temperature loggers in paraffin wax inside limpet shells [1]. Other biomimetic devices, which had previously accurately estimated limpet internal body temperatures [2], were trialled but were either damaged, lost or malfunctioned and therefore the data collected from them was not useable. As we were interested in the relative body and head-end temperatures of differently orientated limpets, not absolute temperatures, the use of paraffin wax [1] to mimic limpet bodies was determined to be sufficient. Limpets were placed on sandstone blocks within a drying oven, which was set to five different temperatures sequentially (23-42ºC). Limpets were also photographed in natural conditions before heating commenced. At each temperature limpets were heated for ~ 30 minutes and then removed from the oven and photographed. The temperature of the limpet shells was measured using the program IRSoft (Version 3.3, Testo AG) and then compared to the temperature recorded by the loggers. The null hypothesis that was there was no significant relationship between shell temperature and the temperature inside the artificial limpets was tested using a linear regression in SPSS (IBM SPSS Statistics 21). A Durbin-Watson test was used to test if the residuals were autocorrelated and a plot of the residuals versus the predicted values was used to check if the assumption of homoscedasticity was met.

There was a significant linear regression between shell temperature and the internal temperature of artificial limpets ($F_{(1,34)} = 4748.04$, $p < 0.05$, $R^2 = 0.993$, Figure 1, Table 1). There was no significant autocorrelation (Durbin-Watson = 2.553) and assumptions of homoscedasticity was met. The following equation derived from this experiment can be used to calculate body (BT) and head-end temperature (AT) : BT or AT = 1.34 + 1.01ST.

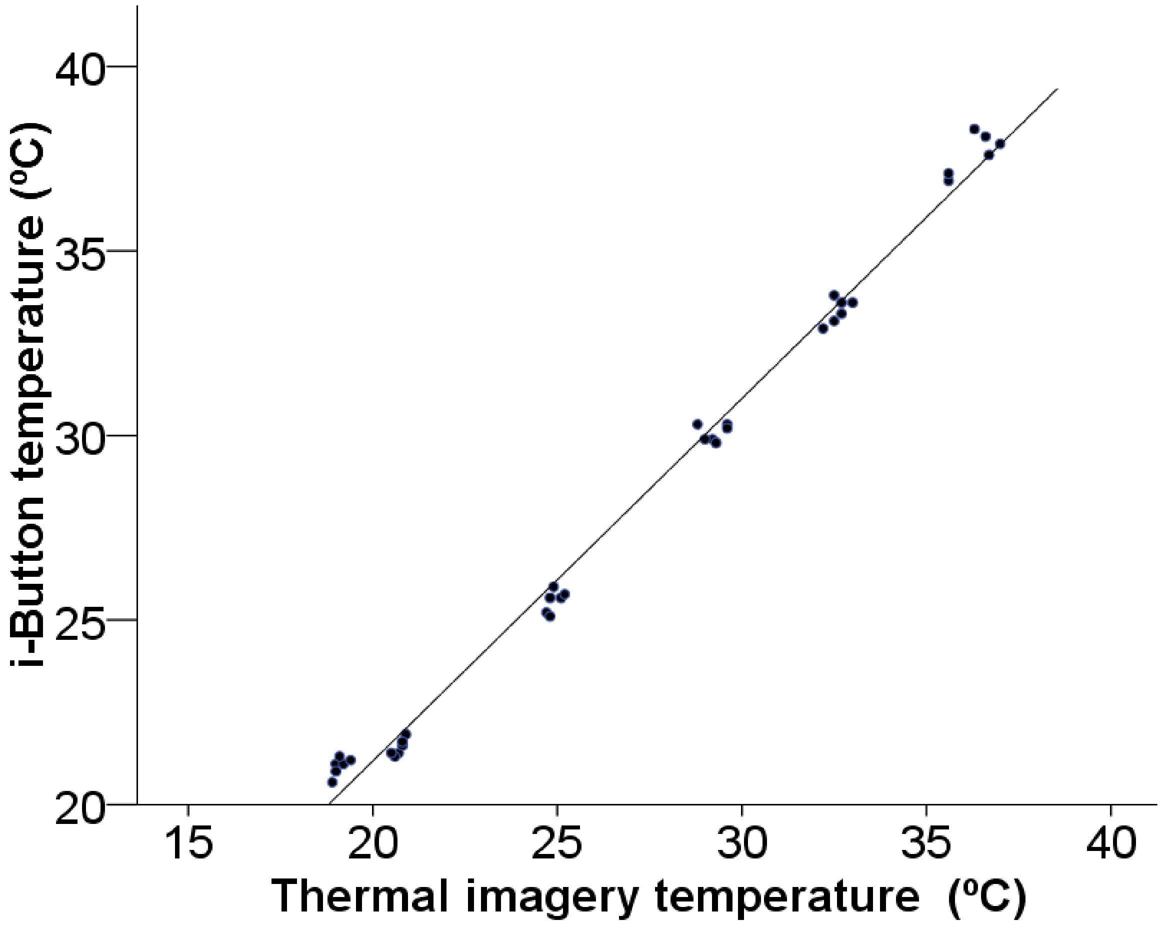

Figure 1. Regression of the temperature of artificial limpets measured using thermal imagery against the temperature measured by iButtons. Line equation: Body Temperature or Head-end Temperature = 1.34 + 1.01 x Shell Temperature. 95% CI has been omitted as it was so close to the main plot as to obscure the figure.

Table 1. Testing the null hypothesis that there is no linear regression between temperatures of artificial limpets measured using thermal imagery and iButtons.

Source	df	MS	F	p
Regression	1	1367.34	4748.05	<0.05
Res	34	0.29		

References

1. Sinclair ELE, Thompson MB, Seebacher F. Phenotypic flexibility in the metabolic response of the limpet *Cellana tramoserica* to thermally different microhabitats. J Exp Mar Biol Ecol. 2006;335(1):131-41. doi: 10.1016/j.jembe.2006.03.010.
2. Lima FP, Wethey DS. Robolimpets: measuring intertidal body temperatures using biomimetic loggers. Limnology and Oceanography-Methods. 2009;7:347-53.
